# A Common Anesthesiology Procedure for a Patient with an Uncommon Combination of Diseases: A Case Report

**DOI:** 10.1155/2012/748748

**Published:** 2012-12-12

**Authors:** Aliki Tympa, Dimitrios Hassiakos, Nikolaos Salakos, Aikaterini Melemeni

**Affiliations:** ^1^1st Department of Anesthesiology, Aretaieion University Hospital, 76 Vas. Sofias Avenue, 11528 Athens, Greece; ^2^1st Department of Obstetrics and Gynecology, Aretaieion University Hospital, 76 Vas. Sofias Avenue, 11528 Athens, Greece

## Abstract

Administering neuraxial anesthesia to a patient with an underlying neurological disease and a combination of four other pathological disorders can be challenging. We report in this paper the case of a 45-year-old woman with neurological deficit due to ischemic brain infarct, multiple sclerosis, antiphospholipid syndrome, and **β**-heterozygous thalassemia that was subjected to abdominal hysterectomy and bilateral salpingoophorectomy under epidural anesthesia for ovarian cancer.

## 1. Introduction

 The choice of anesthetic technique in patients with preexisting neurological disease may be a specific concern for anesthetists [1]. Neurological deficits appearing after spinal or epidural anesthesia have cast additional doubt on the benefit of neuraxial anesthesia in these patients [2, 3]. If administering neuraxial anesthesia to a patient with an underlying neurological disease is puzzling, then the conduct of anesthesia in a patient with preexisting neurological deficit and a combination of four other pathological disorders is undoubtedly challenging. Safely caring for the health, comfort, and quality of life of a patient with neurological disease and several other comorbidities is a difficult task that extends beyond the operating and recovery room. 

## 2. Case Presentation

 A 45-year-old, ASA physical status IV woman (78 kg, 172 cm) with ovarian cancer was scheduled for abdominal hysterectomy and bilateral salpingoophorectomy with epidural anesthesia, after her refusal for general anesthesia. The patient had been successfully subjected to cesarean section under epidural anesthesia four years ago.

 Her clinical history revealed an ischemic infarct of the left frontal lobe since the age of 19, multiple sclerosis since the age of 20, antiphospholipid syndrome (heterozygote for the methylenetetrahydrofolate reductase (MHTFR) gene), and heterozygous *β*-thalassemia. On her admission, normotensive sinus tachycardia (115 bpm), fever and intense lower abdominal pain along with ascites and nausea were noted. The patient presented with 26.5% hematocrit, a platelet count of 465.000/L, and normal bleeding tests. Neurological examination revealed right hemiparesis, dropping of the left corner of the mouth, impaired oropharyngeal function with dysphagia to both solids and liquids, and altered walking gait. The patient had been receiving cinnarizine 10 mg daily, bromazepam 3 mg three times per day, and paracetamol occasionally. 

 In the operating room, with the patient positioned in the left lateral decubitus position, a 18-gauge Tuohy needle was inserted at L_1_-L_2_ intervertebral space; the epidural space was found using the loss-of-resistance technique. A polyamide catheter was easily introduced and placed at the 12 cm catheter mark. A test dose of 3 mL of 2% lidocaine was injected. After catheter placement the patient turned supine and was given 15 mL of ropivacaine 0.75% and 100 *μ*g fentanyl. A sensory block to T_3_ was established 15 minutes later. During the 2-hour surgery, another 5 mL of ropivacaine 0.75% and 10 mL of lidocaine 2% were given as the patient complained of discomfort during peritoneal traction. Entonox inhalation was used to alleviate patients' discomfort during peritoneal traction and abdominal wall closure. At the recovery room, 1/2 an hour after the last epidural dose, the patient experienced severe pain and was administered 8 mL of ropivacaine 0.2% and 2 mg of preservative free morphine followed by 6 mL of ropivacaine 0.375% fifteen minutes later. A sensory block to T_12_ was present 1^1/2^ hours later with no motor block at recovery. 

 Postoperative analgesia with 8 mL ropivacaine 0.2% four times daily, 2 mg morphine twice a day, and rescue dose of 6 mL ropivacaine 0.375% failed and the regimen was replaced by a continuous epidural pump, set to infuse 2 mL of ropivacaine 0.2% and 8 *μ*g fentanyl hourly. The patient also received 3 g of paracetamol daily and triple antiemetic drug combination (metoclopramide 30 mg, 5HT_3_ antagonist 12 mg, and dexamethasone 8 mg). On the second postoperative day, pain was effectively managed with the continuous pump infusion but nausea persisted. Neurological examination revealed no signs of deterioration of the preexisting disease. The epidural catheter was removed on the fifth postoperative day. Nausea was not alleviated despite rigorous treatment. The patient developed paralytic ileus on the sixth postoperative day that resolved spontaneously two days later.

## 3. Discussion

 Neurological deficit, chronic neuropathic cancer pain, antiphospholipid syndrome, and hematological disorders along with the patients' refusal for general anesthesia form a noteworthy clinical profile, challenging for every anesthetist. 

In the present case, we report the anesthetic management and the medical questions raised during the clinical course of a patient with the combination of the abovementioned uncommon diseases. 

 Worsening of neurological symptoms in patients with preexisting neural compromise has always been a fear for the neuraxial anesthesia and analgesia performing anesthetist [1–3]. Disorders of the central nervous system such as multiple sclerosis have historically been a relative contraindication to spinal and epidural anesthesia. Dripps and Vandam [4] were the first to report exacerbation of preexisting neurological disease after spinal anesthesia. Several years later, Brinkmeier et al. [5] provided evidence that an endogenous pentapeptide in patients with multiple sclerosis has a higher blocking efficacy than that of 50 *μ*M of lidocaine and may well contribute to the fast changes of symptoms.

 Epidural anesthesia is commonly thought to be less harmful than spinal anesthesia; however secondary parameters such as local anesthetic neurotoxicity and needle or catheter induced trauma may cause relapse of the dormant underlying neurologic pathology. Furthermore, many perioperative factors such as fever, infection, stress, fatigue, and the operation itself may result in deterioration of the preexisting neural compromise. To this direction, Hebl et al. [1] in their retrospective review of 136 patients with preexisting central nervous system disorders suggest that the risks commonly associated with epidural or spinal anesthesia and analgesia may not be as frequent as once thought.

 The patient has also been diagnosed with antiphospholipid syndrome; an autoimmune disorder characterized by vascular thrombosis. The need for anticoagulant therapy for the patient with antiphospholipid syndrome may prove higher postoperatively and this unanticipated anticoagulation regimen explains the reluctancy in performing neuraxial blockade [6].

 Given the intense postoperative pain experienced by the patient in the recovery room and the ward along with the patients' characteristics, the roles of opioid-induced hyperalgesia and preemptive analgesia come into question. The cancer patient with neurological deficit gathers several potential preoperative risk determinants of postsurgical pain; anxiety, depression, and catastrophising personality along with impaired pain modulation have been postulated as primary causal factors leading to postsurgical pain [7]. The intensity of perioperative pain—which in this case was not negligible—has also been suggested as a key risk factor to postoperative pain [7–9]. Acute opioid-induced hyperalgesia (resulting from the desensitization of antinociceptive pathways) in the perioperative period may be an explanation for the postsurgical pain; opioid-induced hyperalgesia can occur in various, low-dose, high-dose, or maintenance-dose regimens of opioids [7]. Hyperalgesia induced to patients with preexisting neurological deficits and chronic pain is a matter that remains to be elucidated in the future. 

 Preemptive analgesia could probably play a role in the clinical setting of this patient [8–11]; however NSAIDs carry the risk of precipitating thromboembolic events to patients with antiphospholipid syndrome [6] while gabapentin may eventually deteriorate the underlying neurological pathology. Alternative methods of preemptive analgesia including a_2_ agonists or ketamine [10] could have been used to improve analgesia while reducing opioid-related side effects. Of course, the role of preemptive analgesia in patients with preexisting neurological deficit or antiphospholipid syndrome has not been studied, but identification of the problem is the first step to improving outcomes after surgery. 

 Another skepticism that emerges from this case report is whether spinal anesthesia with profound sensory and motor block intraoperatively could have resulted in reduced postoperative pain. Recent studies on spinal anesthesia in patients with preexisting central nervous system disorders report complication-free procedures with patient satisfaction [1]. 

 Although epidural anesthesia to this patient may have been a common final procedure, balancing the risks and benefits of the anesthetic technique and critically questioning the problems raised by her clinical course was a copious work in the hope that definitive conclusions will be made after new prospective studies.
